# Cone photoreceptor definition on adaptive optics retinal imaging

**DOI:** 10.1136/bjophthalmol-2013-304615

**Published:** 2014-04-11

**Authors:** Manickam Nick Muthiah, Carlos Gias, Fred Kuanfu Chen, Joe Zhong, Zoe McClelland, Ferenc B Sallo, Tunde Peto, Peter J Coffey, Lyndon da Cruz

**Affiliations:** 1 National Institute for Health Research, Biomedical Research Centre for Ophthalmology, London, UK; 2 Moorfields Eye Hospital, London, UK; 3 Division of Cellular Therapy, UCL Institute of Ophthalmology, London, UK; 4 Centre for Ophthalmology and Visual Science (incorporating Lions Eye Institute), The University of Western Australia, Perth, Australia; 5 Department of Research and Development, The Reading Centre, Moorfields Eye Hospital, London, UK

**Keywords:** Adaptive Optics, Retinal Imaging, Cone Photoreceptor

## Abstract

**Aims:**

To quantitatively analyse cone photoreceptor matrices on images captured on an adaptive optics (AO) camera and assess their correlation to well-established parameters in the retinal histology literature.

**Methods:**

High resolution retinal images were acquired from 10 healthy subjects, aged 20–35 years old, using an AO camera (rtx1, Imagine Eyes, France). Left eye images were captured at 5° of retinal eccentricity, temporal to the fovea for consistency. In three subjects, images were also acquired at 0, 2, 3, 5 and 7° retinal eccentricities. Cone photoreceptor density was calculated following manual and automated counting. Inter-photoreceptor distance was also calculated. Voronoi domain and power spectrum analyses were performed for all images.

**Results:**

At 5° eccentricity, the cone density (cones/mm^2^ mean±SD) was 15.3±1.4×10^3^ (automated) and 13.9±1.0×10^3^ (manual) and the mean inter-photoreceptor distance was 8.6±0.4 μm. Cone density decreased and inter-photoreceptor distance increased with increasing retinal eccentricity from 2 to 7°. A regular hexagonal cone photoreceptor mosaic pattern was seen at 2, 3 and 5° of retinal eccentricity.

**Conclusions:**

Imaging data acquired from the AO camera match cone density, intercone distance and show the known features of cone photoreceptor distribution in the pericentral retina as reported by histology, namely, decreasing density values from 2 to 7° of eccentricity and the hexagonal packing arrangement. This confirms that AO flood imaging provides reliable estimates of pericentral cone photoreceptor distribution in normal subjects.

## Introduction

In vivo cellular imaging of the human retina has been made possible through the emergence of high resolution adaptive optics (AO) retinal imaging systems.^1^ Prior to the development of AO retinal imaging devices, assessment of ultra-structural features and arrangement of cones was via histology of enucleated globes or biopsy specimens. However, the ex vivo techniques of laboratory histology are limited by artefacts of tissue processing and restrict observations to a single time point.

The advent of AO has led to a steady development of prototype devices over the past 17 years.^2^ These have been based either on confocal scanning laser ophthalmoscope (SLO)^3^ or fundus flood-illumination cameras.^4^ In 1996, Miller and colleagues produced a research prototype fundus camera using monochromatic light with a small field of illumination and a non-coherent laser source. This device enabled the imaging of the cone mosaic in healthy eyes in vivo.^5^ The technique involved fine correction of the subject's astigmatism and defocus with trial lenses.^5^ Further improvement in image resolution was achieved by incorporating an AO system based on a deformable mirror.^6^ This system continuously and automatically compensated for ocular aberration based on feedback from a wavefront sensor^7^ that enabled diffraction-limited retinal imaging. Images from in vivo AO devices have the advantage of no tissue processing artefacts and the ability to carry out serial cone imaging in the same subject.

Histological examination has shown cone photoreceptors to have the following characteristics: A density of approximately 50 000 cones/mm^2^ at 1° temporal to fovea, significant reduction in density from the centre of the retina up to 6.2° (2 mm) and a hexagonal pattern of organisation.^8–10^

The rtx1 adaptive optics camera (AOC) (Imagine Eyes, Orsay, France) uses a flood-illumination camera for image capture. The size and design of the device as well as the recent European regulatory approval (CE mark) allow it to be used in a normal clinical setting. Given this, it is critical to document the ability of the rtx1 AOC to successfully identify cone photoreceptors and understand its limitations. Cone imaging has been described qualitatively in macular disease by Paques and colleagues and others^11–13^ and quantitatively by way of cone density in healthy subjects using the rtx1 AOC.^14^ Crucially though, detailed qualitative and quantitative analysis of the signals in relation to cone matrices such as photoreceptor organisation, cone density and intercone distance in age-matched controls has not been reported. There have been studies on AO SLO prototype systems, which have correlated their images with those from the histology literature.^15^ ^16^ However, these cannot be presumed to imply that the cone signals from the rtx1 AOC images are comparable.

The aim of this study was to assess the feasibility of cone photoreceptor image capture and to analyse the images for retinal photoreceptor parameters in comparison with previous histological and AO imaging data available in the literature.

## Material and methods

### Subjects

Ten healthy volunteers were recruited from the staff of Moorfields Eye Hospital and UCL Institute of Ophthalmology; age range 20–35 years (mean=26 years, SD=3); one male and nine female volunteers. The study protocol was approved by the Moorfields and Whittington NHS Research Ethics Committee and complied with the tenets of the Declaration of Helsinki (2008 Revision).

### Clinical investigations, inclusion criteria

All subjects had a complete eye examination to exclude any ocular pathology or media opacities and to confirm best-corrected visual acuity was 6/6 or better for inclusion in the study. Subjects’ refraction was recorded and ranged from spherical equivalent plano to −6.50 D (mean=−2.50 D). For imaging, low-order aberrations were corrected internally by a telecentric system where necessary. Axial length was measured using an IOLMaster (Carl Zeiss Meditec, Germany) and ranged from 22.08 to 26.02 mm (mean=24.22 mm, SD=1.58 mm).

#### Retinal imaging

##### Scanning laser ophthalmoscopy

**Figure 1:**
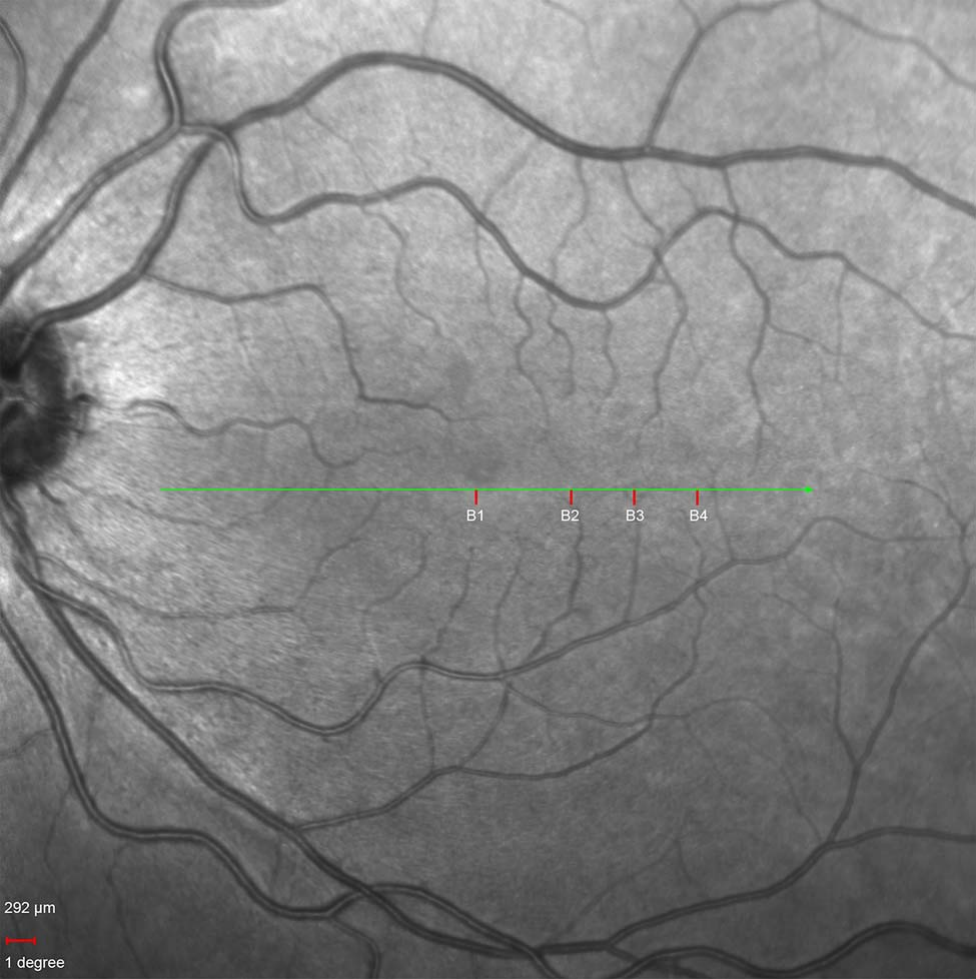
Infrared fundus image of subject B's left eye. B1, B2, B3 and B4 on the image mark the points at 0, 3, 5 and 7°, respectively, at which the adaptive optics retinal images were acquired. Scale bar is 292 μ, equivalent to 1°.

Confocal SLO was performed using a Spectralis SLO (Heidelberg Engineering, Heidelberg, Germany) device. The infrared SLO fundus image obtained was used as a topographical reference for the location of the various eccentricities at which the AO images were acquired (figure 1).

##### AO imaging

Imaging was performed using the rtx1 AOC device through undilated pupils, following 5 min of dark adaptation. This automated en-face reflectance imaging system uses an infrared (wavelength, λ=850 nm) flash for illumination and an AO system consisting of a Shack–Hartmann sensor and a deformable mirror for correcting aberrations. The field of imaging is 4×4° which is equivalent to 1.2×1.2 mm on the retina based on the Gullstrand model eye of axial length 23.0 mm. A set of 40 frames is captured live. During image processing, each of the 40 frames is coregistered and averaged by the internal software provided by the manufacturer. During this process, an image with a resolution of 750×750 pixel (px) is converted to 1500×1500 px. The final image produced based on the axial length of the model eye has a resolution of 0.8 μm/px.

**Figure 2:**
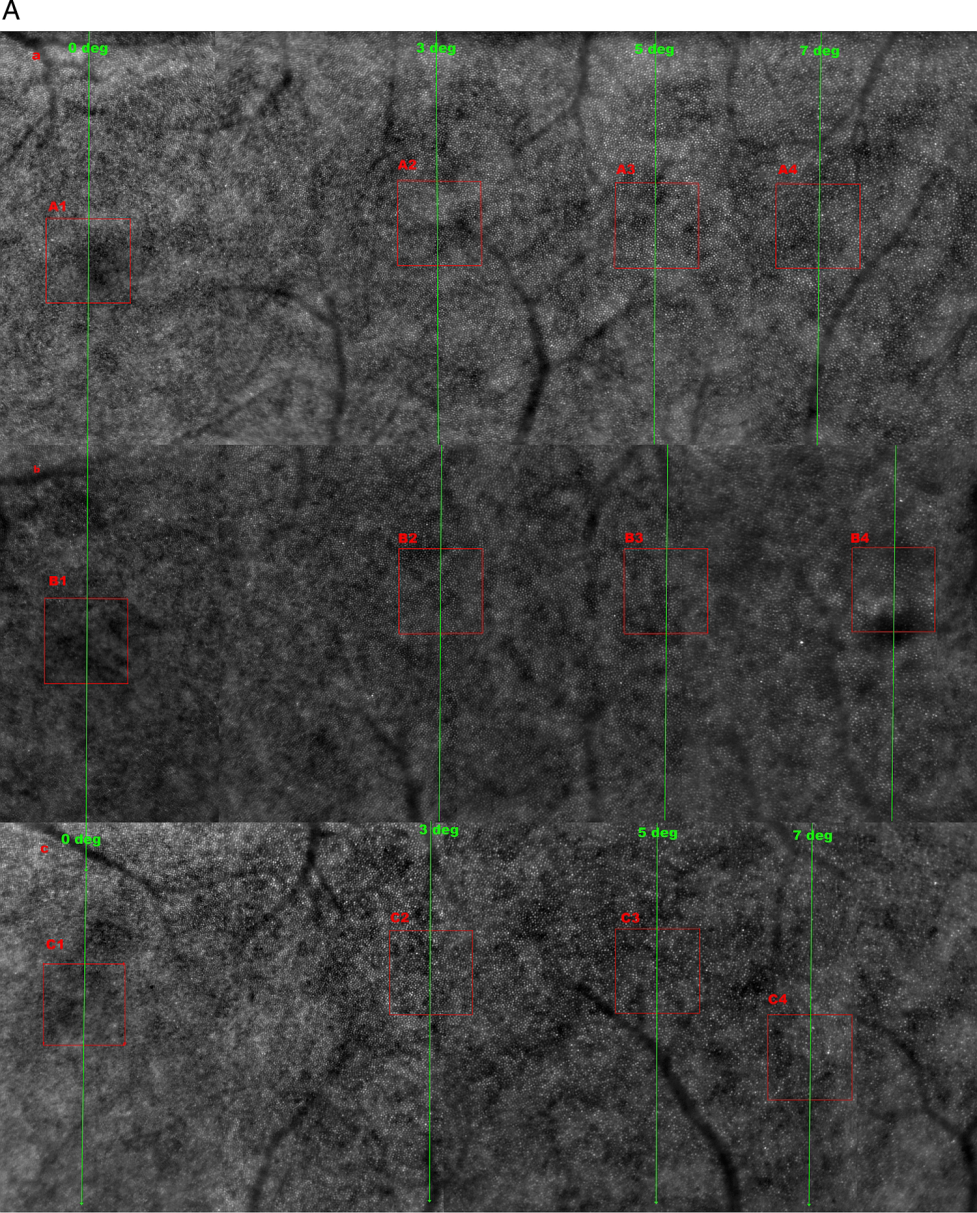

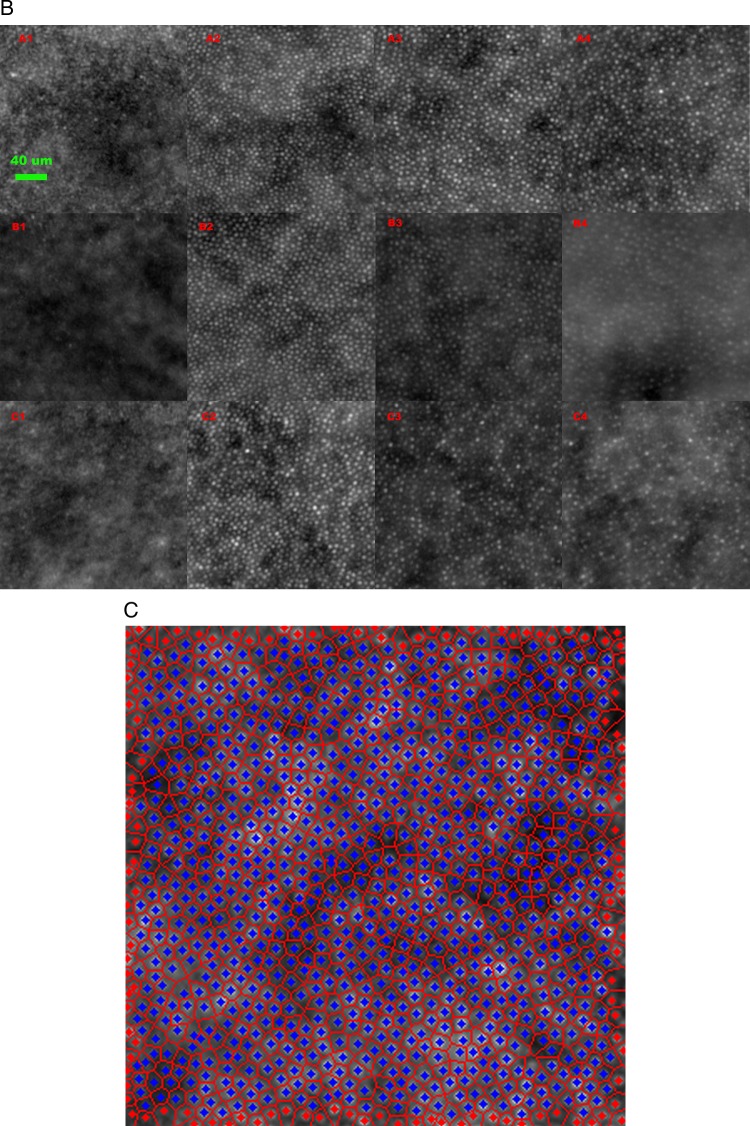
(A) Adaptive optics (AO) retinal image montages of subjects A, B and C from 0 to 7°. This image shows the decreasing cone photoreceptor density with increasing retinal eccentricity. In images A, B and C (1, 2, 3 and 4) correspond to AO imaging at 0, 3, 5 and 7° retinal eccentricities. The cones are clearly visible in the 3, 5 and 7° images, but not at 0°, and this is due to the resolution limit of the rtx1 AO camera being 4 μ and therefore is not able to resolve the highest density of cone packing at the foveola. (B) Magnified areas of the red box from figure 2A of AO images of subjects A, B and C at 0, 3, 5 and 7° retinal eccentricities. The magnified AO images of A2 through A3 to A4, and similarly for B2 to B4 and C2 to C4 clearly show the cone photoreceptors with decreasing density at increasing retinal eccentricity as well as the loss of their packing regularity in A4, B4 and C4. The cone photoreceptors in images A1, B1 and C1 are not discernible due to the highest cone packing density at 0° which is beyond the device's resolution. (C) Voronoi tessellation of subject A's retinal image at 5° retinal eccentricity.

The left eyes of subjects were imaged, although both eyes fulfilled the inclusion criteria. Images were obtained at 5° (∼1.5 mm), temporal to the foveal centre in all study eyes. Three subjects were chosen at random and imaging also performed at 0, 2, 3, 5 and 7° to examine photoreceptor density at multiple retinal eccentricities (figure 2A). The magnified AO retinal images are shown in figure 2B.

#### Image analysis

##### Cone density and packing regularity

Cone mosaic is a two-dimensional variable. The two most common types of matrices used to describe cone mosaics are cellular density and packing regularity. To calculate cellular density, we manually counted cone photoreceptors using an image-processing program (ImageJ, National Institutes of Health, Bethesda, MD, USA). The count was then divided by the area of the retina sampled. Packing regularity was analysed using the following methods:

1. Nearest-neighbour method as described by Wassle and Riemann.^17^
2. Voronoi domain method as described by Shapiro and colleagues.^18^
3. Autocorrelation methods as described by Rodieck^19^ and Cook.^20^
4. Power spectrum method as described by Yellott.^21^

These methods have been previously described for analysis of spatial distribution of rods and cones in vitro and in vivo.^22^ ^23^

##### Automated algorithm cone identification

The retinal images were processed with a customised program coded using MATLAB R2010a (MathWorks Inc, Natick, Massachusetts, USA), similar to a previously described method by Li and Roorda.^15^ The manufacturer's counting software was not used, as it could not perform the count on the sampled windows used in this study. The acquired images were converted to 8-bits and cropped to 300×300 px (∼240×240 μm) sampling window.

**Figure 3:**
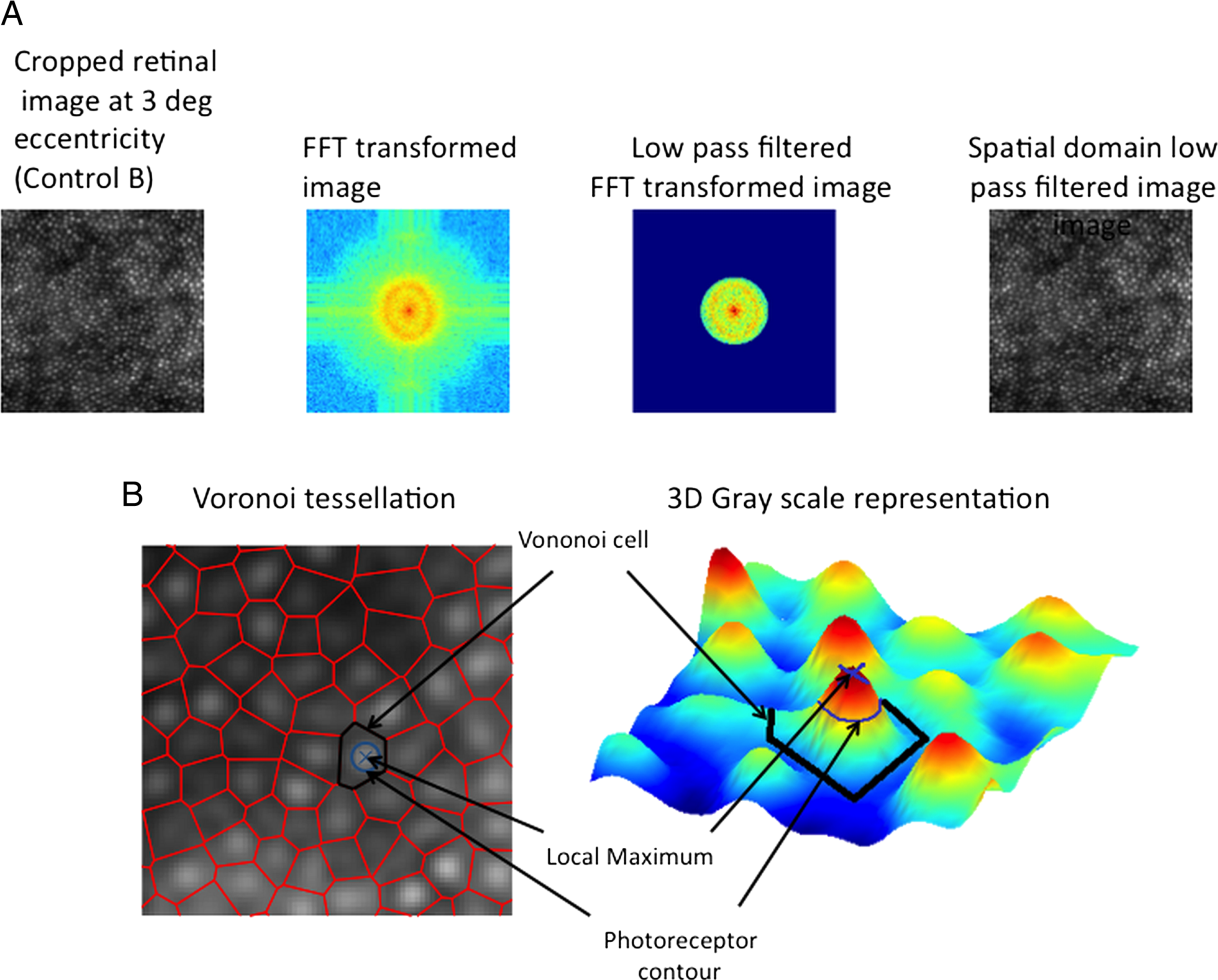
(A) Spatial frequency technique for processing of adaptive optics images. (B) Voronoi cell analysis for inter-photoreceptor distance approximation.

A low-pass filter was applied prior to the automated counting algorithm for all subjects at all eccentricities. The number of spurious peaks were reduced by transformation to frequency domain using fast Fourier transform and preprocessed with a low-pass filter before converting them back to the spatial domain (figure 3A). The regional maxima of the photoreceptors’ centres were computed using an 8-connected neighbourhood. A Delaunay triangulation with its corresponding Voronoi tessellation was calculated resulting in a set of edges linking all the maxima points. Average number of photoreceptors surrounding each cone was calculated by determining the average number of edges originating from each maximum point. The average distance of all the edges was taken as the average inter-photoreceptor distance. The photoreceptor size was approximated by measuring the area of the joint pixels surrounding each maximum, with a greyscale value greater than the average between a peak and its local baseline. The grey scale value of the local baseline is calculated as the average value of the pixels that form the edges of the Voronoi cell of a given peak (figure 3B). The equivalent diameter of a circle with the same area as the one calculated is taken as the diameter of the photoreceptor. Inter-photoreceptor distance was therefore measured by automated technique from the centre of one photoreceptor to its neighbours.^15^

##### Automated and manual cone counting

Automated and manual counting was performed using 10 high quality images of controls at 5° retinal eccentricity, with a central sampling area 300×300 px (equal to the central 240×240 μm). The observer was masked to the identity of the subjects during this process.

The density of photoreceptors with varying retinal eccentricity was also calculated using both manual and automated counting techniques in three subjects at 2, 3, 5 and 7° retinal eccentricities. The AO images captured at 0° were not included as part of either counts as the device was unable to resolve any retinal structure less than 4 μm, as noted in figure 2A,B.

##### Voronoi domain analysis

Voronoi tessellation was performed on the AO retinal images of the 10 subjects following cone identification by the automated algorithm. The percentage of cone photoreceptors showing optimal hexagonal (n=6) tiling as well as 5- and 7-sided (n±1) organisation was calculated for each of the 10 images. We manually excluded the polygons on the edges of the image to avoid any bias to result (figure 2C. Voronoi tessellation of subject A's retinal image at 5° retinal eccentricity). Voronoi quantification was also completed for the three subjects at the 2, 3, 5 and 7° retinal eccentricities.

##### Power spectrum analysis

Spatial regularity (hexagonal packing) of photoreceptors is known to result in a ring structure in the power spectrum of a retinal image.^15^ This analysis was performed in our study (figure 3A).

## Results

### Cone density at 5°

Cone photoreceptor density at 5° measured using the automated algorithm was 15.3±1.4×10^3^ cones/mm^2^ (mean±SD, n=10) and manually 13.9±1.0×10^3^ cones/mm^2^ (mean±SD).

### Cone density with varying eccentricity

The density of photoreceptors decreased with increasing retinal eccentricity temporally as noted in figure 2A,B. The paired mean cone density from manual and automated counts at 2, 3, 5 and 7° were 26.5×10^3^ and 24.2×10^3^ cones/mm^2^, 19.5×10^3^ and 20.8×10^3^ cones/mm^2^, 13.8×10^3^ and 15.6×10^3^ cones/mm^2^ and 11.2×10^3^ and 12.9×10^3^ cones/mm^2^, respectively. Details of the densities of each of the three subjects (A, B and C) at the eccentricities from the manual and automated count results are shown in tables 1 and 2.

**Table 1:** Manual cone density counts at increasing retinal eccentricities

	Manual cone density counts (×10^3^ cones/mm^2^)			
Subjects	2°	3°	5°	7°
A	26.8	21.7	15.4	12.6
B	25.4	19.3	13	10.6
C	27.3	17.6	12.9	10.4
Mean	26.5	19.5	13.8	11.2

**Table 2:** Automated cone density counts and inter-photoreceptor distances at increasing retinal eccentricities

	Automated cone density counts (×10^3^ cones/mm^2^) (Inter-photoreceptor distance) (in μm)			
Subjects	2°	3°	5°	7°
A	24.3 (6.8)	23.1 (7.0)	17.6 (8.0)	15.6 (8.6)
B	24.7 (6.7)	20.2 (7.4)	14.5 (8.7)	10.8 (9.9)
C	23.5 (6.9)	19 (7.7)	14.8 (8.5)	12.2 (9.5)
Mean	24.2 (6.8)	20.8 (7.4)	15.6 (8.4)	12.9 (9.3)

### Inter-photoreceptor distance

Inter-photoreceptor distance measurement calculated by automated technique, from the centre of one photoreceptor to the neighbouring ones, as described by Li and Roorda,^15^ had a range of mean inter-photoreceptor distance of 7.9–9.3 µm at 5° for the healthy subjects. The overall mean of the 10 controls at 5° eccentricity was 8.6±0.4 μm (mean±SD).

The inter-photoreceptor distances for the three subjects imaged at 2, 3, 5 and 7° are also recorded in table 2.

### Voronoi quantification

The hexagonality of cone photoreceptor (n=6) tiling for the 10 subjects at 5° retinal eccentricity ranged from 45% to 55%, mean of 49%. With the inclusion of both the 5- and 7-sided organisation (n±1), the percentage range was 92%–98% with a mean of 95%.

The average proportion of hexagonal tiling in the three subjects was 51% at 2°, 55% at 3°, 50% at 5° and 43% at 7°. The inclusion of 5-, 6- and 7-sided organisation was 95%, 98%, 94% and 90% at 2, 3, 5 and 7°, respectively, for the three subjects.

### Packing regularity of cones

**Figure 4:**
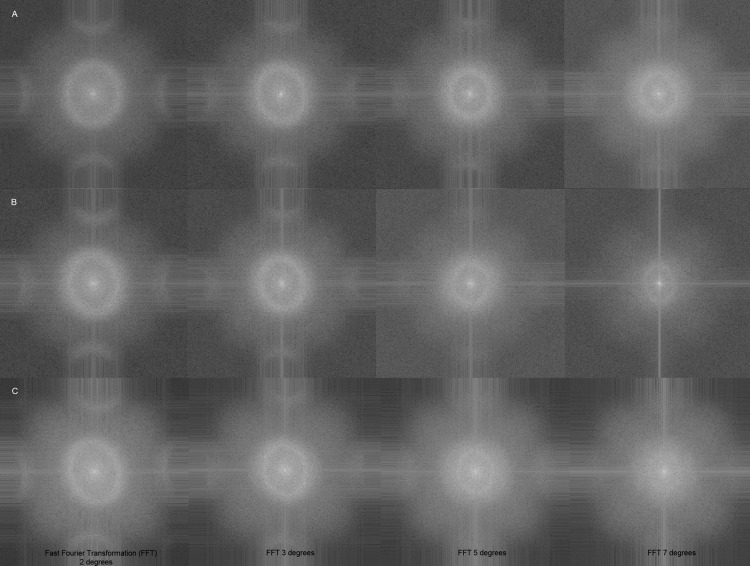
Fast Fourier transformation at 2, 3, 5 and 7° retinal eccentricities of subjects A, B and C.

The regularity of hexagonal cone arrangement has been demonstrated by spatial frequency analysis. Figure 4 shows the classic ring structure in the power spectrum at 2, 3 and 5°, which are synonymous with spatial regularity at these eccentricities but beyond 7° the ring is just visible in two subjects and not in the other. This is due to the decreasing degree of regularity beyond 5°.

## Discussion

We have shown that retinal imaging in healthy eyes using the rtx1 AOC is feasible and has enabled assessment of cone characteristics. The density of speckled signals following manual and automated counting correlated well with the data from published retinal histology literature. Inter-photoreceptor distance and packing regularity of speckled signals also suggest that these arise from cones.

**Figure 5:**
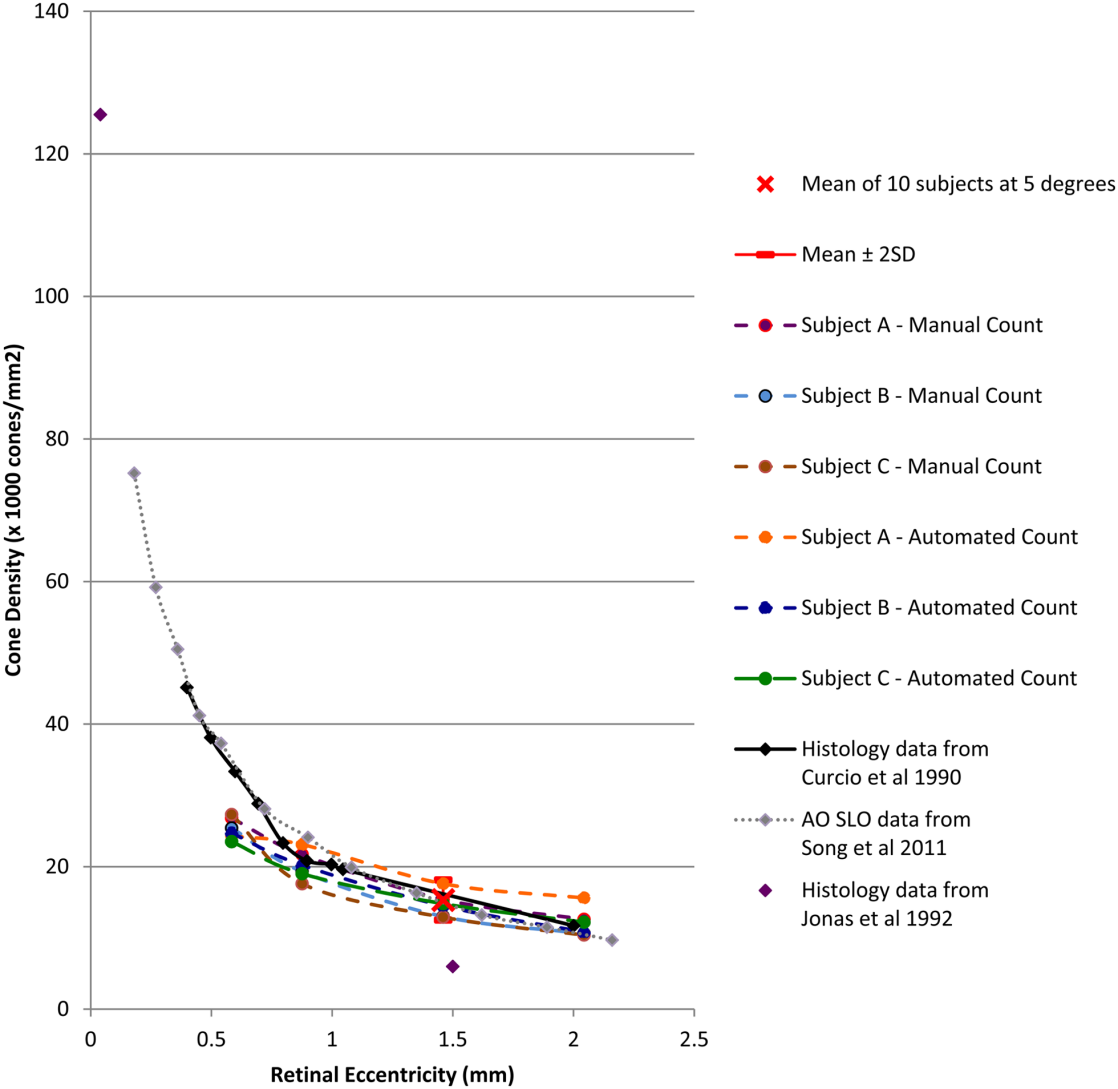
Cone density versus retinal eccentricity for manual and automated count dataset of the three subjects at 2, 3, 5 and 7° and mean of 10 subjects at 5°—plotted on graph with histology data from Curcio *et al*,^8^ Jonas *et al*^9^ and adaptive optics scanning laser ophthalmoscope data from Song *et al*.^16^ Exponential pattern was noted.

The photoreceptor densities (cones/mm^2^ (mean±SD)) at 5° (1.46 mm) temporal to fovea were 15 316±1405 (automated) and 13 901±962 (manual). This correlates closely to that from retinal histology studies on donated healthy human eyes by Curcio and colleagues of 16 188 cones/mm^2^, extrapolated from the graph at 1.46 mm (figure 5). Our AOC data also correlate closely to that from an AO SLO study by Song and colleagues of 16 300±2850 cones/mm^2^ (mean±SD) at retinal eccentricity of 1.35 mm.^8^ ^16^ Our automated count is also similar to that found in a previous study by Lombardo *et al*^14^ on the same device which found mean cone density at 1300 µm (4.45°) eccentricity to be 14 198±2114 cones/mm^2^. The concordance of cone densities between all of these studies is clearly visible in figure 5, where cone density is plotted against retinal eccentricity. The only disparate figure in the literature was from Jonas *et al*^9^ where cone density at 1.5 mm (∼5°) retinal eccentricity was recorded as 6000 cones/ mm^2^. This was less than half of all other studies. It is possible that this is due to the inclusion of eyes up to 90 years old, thereby not being age-matched and indicating cone loss later in life.

Decreasing cone density with increasing retinal eccentricity at 2, 3, 5 and 7° temporally was confirmed by both manual and automated counting. The rate of change followed the Curcio graph well including the gradient decreasing at greater eccentricities (figure 5). The change was confirmed by the inverse relationship noted between inter-photoreceptor distance and increasing eccentricities as indicated in table 2. This provides an internal validation for the automated algorithm calculation method. The automated algorithm does have some limitations: the range of inter-photoreceptor distance we obtained at 5° for each of the healthy subjects was 7.9–9.3 µm. This was close, but not equivalent, to the histology measurement data of 6–8 µm. However, the histology data from Curcio and Sloan^24^ looked at the minimum inter-photoreceptor spacing between the cones at eccentricity greater than 1 mm. We were unable to measure from the same range, as the noise in the system would create an artificially low minimum inter-photoreceptor distance. Furthermore, with the automated counting algorithm there were false positives at higher eccentricities in some participants. This was due to the noise in the image and therefore we manually selected and consistently applied a low-pass filter at all the retinal eccentricities when using the automated system.

Possibly the most compelling cone related feature of the AO images we have observed is the packing pattern. The regularity of the cone matrix was confirmed using the spatial frequency technique. The power spectrum ring was shown at 2, 3 and 5°, though to a lesser degree at 7° (figure 4). The rings decrease in intensity with eccentricity that is consistent with histological findings of significant decrease in cone density from around 1.4 mm (equal to approximately 5°) as noted by Curcio and Sloan.^24^ The consequent increase in rod photoreceptors at this eccentricity begins to disrupt the orderly packing. We also demonstrated regular hexagonal ordering of cones using the Voronoi method. Li and Roorda^15^ had previously demonstrated this hexagonal photoreceptor packing in 2007 using Voronoi domain analysis with their AO SLO prototype.

We studied the age group of 20–35 year olds but did not have a sufficient sample size or age range to analyse the effect of age. There was conflicting evidence in the literature concerning the effect of age. Gao and Hollyfield^25^ did not find any differences in foveal cone densities in donor eyes ranging from 20 to 90 years, while Song *et al*^16^ noted a reduction in cone density at the fovea with increasing age in their AO study.

The sampling area we chose was considerably larger than most of those quoted in the literature. We decided to use a larger sampling window of 240×240 µm to reduce measurement error. Work from the Carroll lab on an AO SLO device found that with decreasing window size, the error rate for cone density measurement increased.^26^ Most studies used a window of around 50×50 µm. These included histology studies such as that of Hirsch and Miller^27^ who used a window of 56×56 µm and a recent in vivo imaging study using AO SLO by the Burns and colleagues which demonstrated good reproducibility in cone density count, with an area of 50×50 µm on a subject imaged twice in 6 months at the same retinal locus.^16^

The two studies by Lombardo *et al*,^28^ were carried out to assess cone density as a function of eccentricity^14^ and symmetry between the two eyes in healthy subjects, but did not assess all the features crucial to confirm that the signals being studied by the device are from cone photoreceptors.

Curcio *et al*^8^ noted that at 1.3–1.4 mm (approximately 5° temporal to fovea), cones were larger and circular in shape and that rods encircle these cones. The areas of darkness and indistinct reflections in-between the cone reflections in our images are most likely to be rods. The reason we are unable to delineate the rods is that the rtx1 AO device has a resolution of only up to 4 µm. This study addresses all aspects which are crucial in defining and confirming the cone photoreceptor matrices on this AOC. Curcio and colleagues^8^ found that in two human donor eyes, photoreceptor diameter at fovea was 1.6 and 2.2 µm respectively. This accounts for why foveal cone imaging was not possible with this device. Future devices will need to significantly improve in order to resolve the fine and closest packing of cone photoreceptors at the fovea.

## Conclusions

By studying photoreceptor matrices, we have been able to demonstrate that the signals captured by the rtx1 AOC are most likely due to the cone photoreceptors. Furthermore, these cone reflectance images correlate quantitatively with accepted retinal histology findings from the literature.^8^ ^10^ ^24^

It is likely that AO based devices and photoreceptor imaging will play a part in the future diagnosis and monitoring of retinal diseases and treatments. The reproducibility of the images and the consistency of quantification in disease states will need to be confirmed before the full potential of this device as a clinical investigation tool can be confirmed.
